# Evidence for the adaptation of protein pH-dependence to subcellular pH

**DOI:** 10.1186/1741-7007-7-69

**Published:** 2009-10-22

**Authors:** Pedro Chan, Jim Warwicker

**Affiliations:** 1 Faculty of Life Sciences, University of Manchester, Michael Smith Building, Oxford Road, M13 9PT, UK

## Abstract

**Background:**

The availability of genome sequences, and inferred protein coding genes, has led to several proteome-wide studies of isoelectric points. Generally, isoelectric points are distributed following variations on a biomodal theme that originates from the predominant acid and base amino acid sidechain pKas. The relative populations of the peaks in such distributions may correlate with environment, either for a whole organism or for subcellular compartments. There is also a tendency for isoelectric points averaged over a subcellular location to not coincide with the local pH, which could be related to solubility. We now calculate the correlation of other pH-dependent properties, calculated from 3D structure, with subcellular pH.

**Results:**

For proteins with known structure and subcellular annotation, the predicted pH at which a protein is most stable, averaged over a location, gives a significantly better correlation with subcellular pH than does isoelectric point. This observation relates to the cumulative properties of proteins, since maximal stability for individual proteins follows the bimodal isoelectric point distribution. Histidine residue location underlies the correlation, a conclusion that is tested against a background of proteins randomised with respect to this feature, and for which the observed correlation drops substantially.

**Conclusion:**

There exists a constraint on protein pH-dependence, in relation to the local pH, that is manifested in the pKa distribution of histidine sub-proteomes. This is discussed in terms of protein stability, pH homeostasis, and fluctuations in proton concentration.

## Background

The post-genomic era allows many basic questions to be addressed, such as the nature of the biological components [1], control of expression levels for these components [2], their interaction networks and dynamics [3], and the ultimate realisation of metabolic function from the parts [4]. Even for proteins, the most studied nodes of biological interaction and function, there is much to discover about how form relates to function. Differences in the characteristics of amino acid sidechains, and in the stoichiometry of their incorporation into proteins, means that physico-chemical properties of proteomes and sub-proteomes can be variable. Several reports of proteome-wide properties have appeared. Features studied include amyloidogenic potential and biological context [5], propensity for disorder and protein degradation [6], amino acid composition and gene expression [7], protein targeting and N-terminal features [8], and the inclusion of physico-chemical properties into proteome browser resources [9].

Protein isoelectric point (pI) can be conveniently estimated from amino acid sequence. Three-dimensional structure gives rise to charge interactions that are important in considering protein folded state stability, but generally give small changes in pI compared with linear sequence [10]. Several groups have looked at computed proteome-wide pI distributions, with the outcomes falling into two overall categories. First, there has been discussion of the form of the pI distribution with pH [11-13], and demonstration that a general bimodality results from the predominant acidic and basic pKas of the Asp/Glu and Lys/Arg sidechains. Second, the relative populations of peaks (acidity *versus *basicity, or pI bias) has been studied with respect to organism environment and taxonomy, and subcellular location. It has been proposed that average pI correlates with growth temperature for orthologues [14], and with bacterial growth conditions [15], that pI bias correlates with taxonomy [16], and that pI distribution varies according to compartmentalisation within the *Arabidopsis *chloroplast [17]. Other work indicates that, for the most part, pI distributions are not correlated with subcellular location or taxonomy [18]. Amongst these varying conclusions, observations reinforced by multiple reports are that: individual protein pIs tend towards less extreme values for longer sequences, as a result of sampling statistics of acidic/basic amino acids [16,19]; subcellular proteome pIs may give net charge at environmental pH to mitigate against protein aggregation [18,20]; smaller proteomes tend to be more basic [15,16]. This last trend is particularly evident for the small proteomes of intracellular parasites, and does not appear to be fully explicable in terms of genome AT bias. Processes suggested to underlie the trend include adaptation to environmental constraints, such as elevated host pH [15], and differences in the rate of accumulation of mutations (higher in intracellular organisms than free-living ones) [16].

3D structures are known for many proteins, and may be modelled for many more [21]. Structure can be used to predict physico-chemical properties, which in turn can aid understanding of function or environmental adaptation, for example comparing proteins from mesophiles and extremophiles [22] or distinguishing Enzyme Commission classes for enzymes [23]. Charges contribute to protein stability, evident from simple geometric analysis [24]. The role of ionisable groups has been studied extensively, with regard to both protein stability and solubility [25], and in terms of specific functionality, such as proton buffering by hemoglobin [26]. Computational models of charge interactions [27] can be applied across databases, looking for example at predicted ionisations of amino acids [28]. Varying degrees of model complexity have been introduced, and are assessed through agreement with experiment for properties such as pKas, the pH-dependence of folding energy, and mutational effects. We have found that a relatively simply model for charge interactions captures the properties of surface ionisable groups [29], whereas more detailed accounting of a protein/water interface is required for substantially buried groups [30]. Since the great majority of ionisable groups lie at the surface, the simplified method is appropriate for application to wide-scale structural proteomics, so long as detailed questions are not asked of the more buried, typically catalytic site, ionisable groups. This has been shown in a previous study, where we focussed on comparing isoelectric points predicted from sequence and structure [20]. It was found that predicted pI, averaged over the protein structures in a subcellular compartment, tends to lie away from the subcellular pH, consistent with a role in mitigating against isoelectric point aggregation. The subcellular average of the pH at which proteins are predicted to be most stable appeared to be a closer match to subcellular pH, than was the average pI. This preliminary observation, which is consistent with other computational work finding that the predicted pH of maximal stability can be quite different to the pI [31], is now investigated in detail. We find that the (pH-dependent) maximum in protein stabilisation relates to modulation of histidine pKas by 3D interactions. These residues are largely at the surface and not recognised individually to be of primary functional importance, and yet their cumulative properties associate with subcellular pH. We discuss the physiological context for this result, including pH homeostasis, pH sensing and stochastic effects.

## Results and Discussion

### pH-dependence of stability and subcellular pH

**Figure 1:**
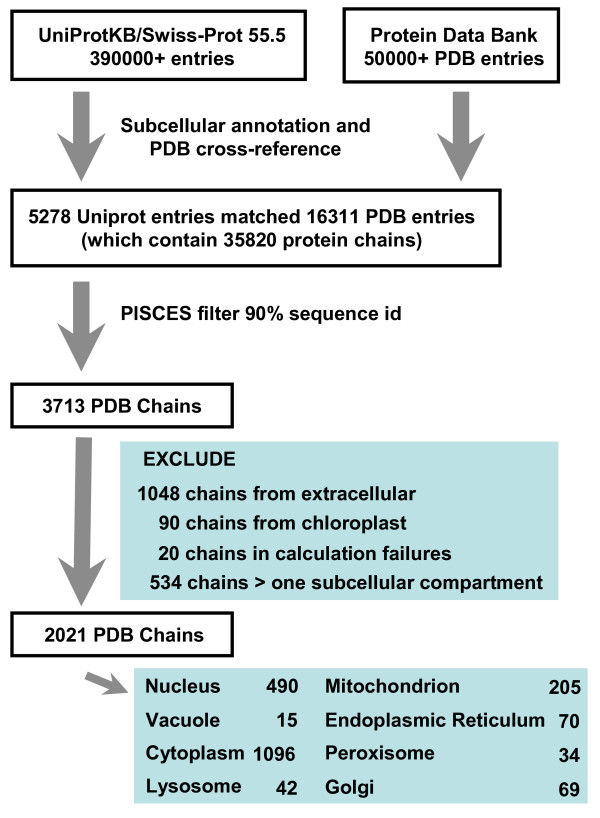
**Construction of the protein structural dataset**. UniProt and the PDB were used to cross-reference subcellular annotation and structure, with filtering for sequence identity and structure quality to give a set of protein chains for calculation. See the Methods section.

**Figure 2:**
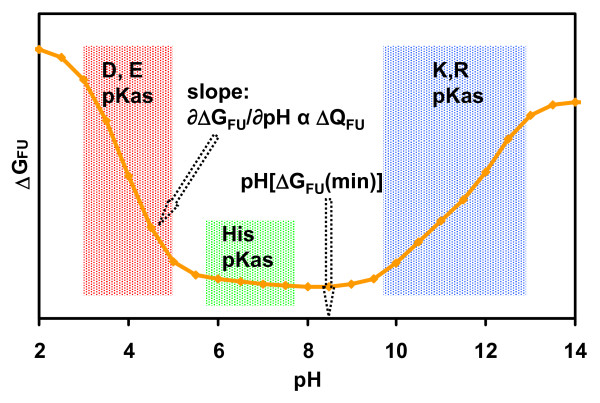
**Major ionisation zones in the pH-dependence of protein stability**. A schematic diagram of the major ionisation regions in a plot of folding stability (ΔG_FU_) versus pH. Properties describing this pH-dependence, in the notional case of no protein unfolding over the pH range, are shown. Cysteine and tyrosine have been omitted from this figure since they are mostly unionised at physiological pH.

A dataset of protein structures annotated by subcellular location was constructed as described in the Methods section (Figure 1). Figure 2 illustrates the major ionisation regions for proteins on a schematic plot of the pH-dependence of folding energy (ΔG_FU_, the difference between the folded (F), and unfolded (U), states). Acidic and basic titrations underpin the generally bimodal pI distributions observed for proteins [32], since the numbers of (Asp + Glu) with acidic pKas, or (Lys + Arg) with basic pKas, normally exceed the number of His, which ionise in the central pH range. While the balance of (Asp + Glu) and (Lys + Arg) mostly determines pI, it follows from the proportionality between ∂ΔG_FU_/∂pH and ΔQ_FU _(the difference in net charge between folded and unfolded forms) [33], that the pH-dependence of stability in the central pH range is determined largely by histidine ionisation (Figure 2). Further, this slope (although small when histidine content is low) is generally negative or positive according to whether the protonated state of histidine is stabilised (ΔQ_FU _positive) or destabilised (ΔQ_FU _negative) in the folded protein. This in turn determines whether the pH value at minimal ΔG_FU _(pH [ΔG_FU_(min)], Figure 2) is towards the acidic or basic titration block, again yielding a generally bimodal distribution.

**Figure 3:**
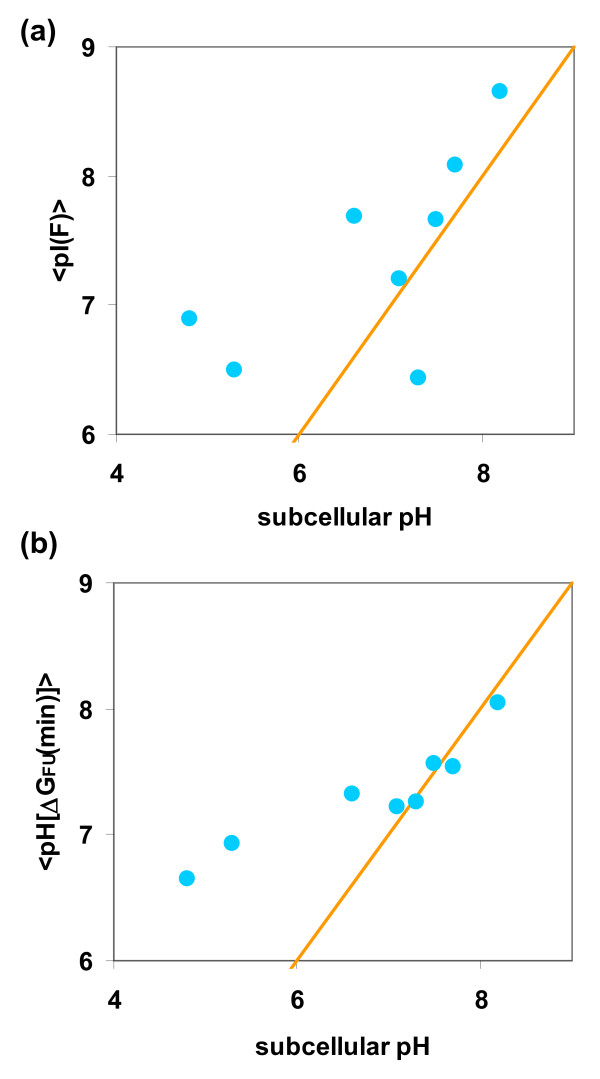
**Subcellular pH and pH-dependent properties**. **(a) **The average over subcellular compartments of predicted folded form pI, plotted against subcellular pH, with R^2 ^= 0.44. **(b) **The pH of maximal folded state stability, averaged over proteins for each subcellular location, is plotted against subcellular pH, R^2 ^= 0.84. For both panels, the line of property = pH is marked (rather than the best fit line).

Isoelectric and pH-dependent properties were calculated and examined for correlation with each other and with the measured environmental pH values (Table 1). The average across each subcellular compartment of the pH at minimal ΔG_FU_, denoted <pH [ΔG_FU_(min)]> correlates better with subcellular pH than do the analogous averages for pI, <pI(F)> and <pI(U)> (see also Figure 3), although none of these properties matches subcellular pH across the entire range. Table 1 also shows the correlations when calculations are repeated with histidine ionisations removed. Here, only average isoelectric properties and subcellular pH are correlated, demonstrating that histidine ionisation lies behind the correlation of <pH [ΔG_FU_(min)]> with subcellular pH (Figure 2). Further, histidine location rather than composition is implicated, since neither subcellular pH nor <pH [ΔG_FU_(min)]> correlate with the subcellular averaged ratio of histidine to other charged amino acids, <His/(Acid+Base)> (Table 1). The nature of pH [ΔG_FU_(min)] *versus *His/(Acid+Base) for individual proteins (not shown) is that more acidic or basic pH [ΔG_FU_(min)] map to higher His/(Acid+Base), while lower His content maps to a relatively underpopulated central zone of pH [ΔG_FU_(min)], bearing out the schematic indications of Figure 2.

**Table 1:** Correlations between calculated properties and subcellular pH

***First property***	***Second property***	***R*^2^*(with His)***	***R*^2 ^*(without His)***
Subcellular pH	<pI(F)>	0.44	0.47
Subcellular pH	<pI(U)>	0.50	0.50
Subcellular pH	<His/(Acid+Base)>	0.02	n/a
Subcellular pH	<pH [ΔG_FU_(min)]>	0.84	0.00
<pH [ΔG_FU_(min)]>	pI(F)	0.70	0.03
<pH [ΔG_FU_(min)]>	pI(U)	0.74	0.01

<pH [ΔG_FU_(min)]>	<His/(Acid+base)>	0.02	n/a

Squares of correlation coefficients are given. Without His refers to calculations with histidine ionisable groups removed.

### Histidine pKas and subcellular location

**Figure 4:**
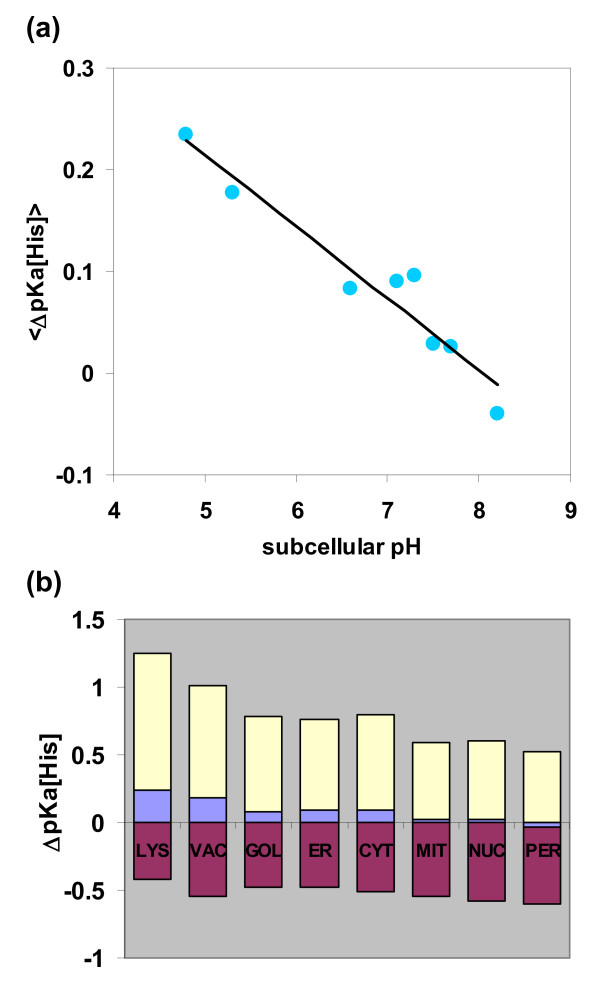
**Histidine and subcellular pH**. **(a) **Predicted ΔpKa per histidine imidazole, averaged over histidines in each subcellular location, is plotted against subcellular pH. The line of best fit is drawn (R^2 ^= 0.92). **(b) **For each subcellular compartment, the average of calculated ΔpKa per histidine is shown, and the 5% and 95% ranked values for ΔpKa in each compartment. LYS, lysosome; VAC, vacuole; GOL, golgi; ER, endoplasmic reticulum; CYT, cytoplasm; MIT, mitochondrion; NUC, nucleus; PER, peroxisome.

Histidine pKa deviations from the model compound value, obtained in the pH-dependence calculation and averaged for each protein, correlate well with <pH [ΔG_FU_(min)]> (R^2 ^= 0.99, not shown). In Figure 4(a), histidine pKa deviations are plotted against subcellular pH, again with good correlation. These are now averaged per histidine, <Δ pKa [His]>, since the protein-specific condition implicit in pH [ΔG_FU_(min)] is lost. Figure 4(b) shows compartment-specific <ΔpKa [His]> with the ranges observed, using the 5% and 95% ranked ΔpKa [His] values within each location. Variation across the subcellular averages is much smaller than the variation of histidine ΔpKas. We have investigated previously whether calculations of <pH [ΔG_FU_(min)]> change substantially upon the inclusion of more a detailed charge interaction scheme, or a model for residual charge interactions in the unfolded state [20]. Although pKas can be perturbed in the U form [34,29], it was found that the effect of these modelling adjustments on <pH [ΔG_FU_(min)]> was small [20]. Furthermore, in the current work, we looked at a sequence-based U form model for charge interactions, with nearest neighbour pairs dominating. If this were to yield correlations with subcellular pH, then the analysis would not be restricted to protein structures. However, the U form model proved too simple, yielding relatively poor correlations (e.g. R^2 ^= 0.29 between <ΔpKa [His]> and subcellular pH), and this line of enquiry was not pursued further.

**Figure 5:**
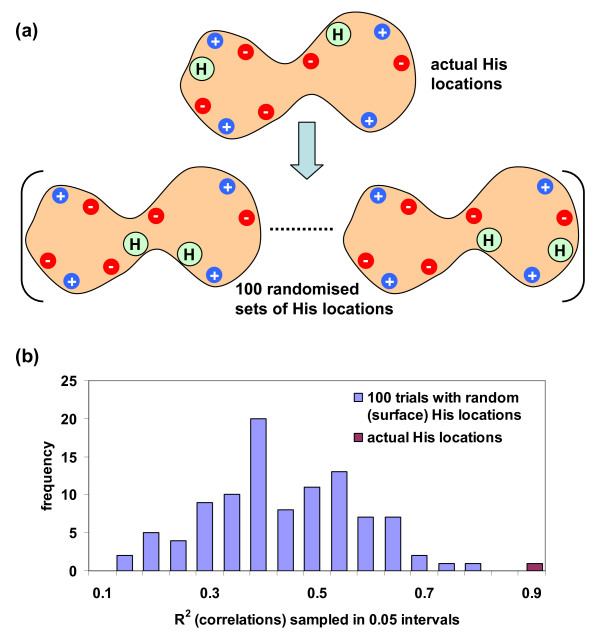
**Histidine location and subcellular pH**. **(a) **Schematic diagram showing randomisation of histidine locations (green circles in the shaded protein shape), with all proteins in the dataset sampled 100 times in this scheme. Background negative (red) and positive (blue) charges are not moved. **(b) **Correlations (given as R^2^) sampled over the 100 randomisations and compared with the actual value (R^2 ^= 0.92). These correlations are between subcellular pH and computed average ΔpKa per histidine (in each subcellular compartment), where the ΔpKa calculations follow the simplified scheme described in Methods.

Having established that the predicted and averaged ionisation properties of histidine sidechains are strongly correlated with subcellular environment, but also bearing in mind that average isoelectric points show some correlation, we investigated further the role of histidine positioning in protein structures. Figure 5(a) illustrates a scheme in which, for each protein, acid and base charges other than histidine are fixed and the ionisable groups of histidine explore alternate surface locations (see also the Methods section). One hundred passes were made through the entire dataset, randomising the location of histidine sidechain charge for each protein and recalculating ΔpKas. In order to make these computations feasible, estimates of ΔpKa from full Monte Carlo sampling were substituted by summation of acid/base interactions at each histidine site, assuming protonated bases and deprotonated acids. For the set of non-randomised proteins, this procedure gave the same R^2 ^(0.92) for <ΔpKa [His]> versus subcellular pH, as did the results of Monte Carlo sampling displayed in Figure 4(a), and the <ΔpKa [His]> values themselves correlated with R^2 ^= 0.999 between the two calculations. Figure 5(b) shows that the correlation with subcellular pH, for calculations with the real distribution of histidine ionisable groups, exceeds that for the randomisations. The net charge of a protein can influence His ΔpKa, simply due to an environment weighted towards positive or negative charge, i.e. positive overall destabilises histidine protonation leading to negative His ΔpKas, and a negatively charged background stabilises His protonation, giving positive His ΔpKas. Indeed, the net charge has some correlation with subcellular pH, as seen in Table 1 and Figure 3(a), whereas Figure 5 demonstrates that an additional element of correlation with subcellular pH is attributable to histidine location, beyond the net charge background (which remains constant in the randomisations). Reinforcing this conclusion, R^2 ^for the correlation between the average of net charge on a protein (excluding histidine), over subcellular location, and subcellular pH is 0.42, substantially less than that for <ΔpKa [His]> and subcellular pH of 0.92. Next we consider the physiological context for our observations.

### Relevance of correlation between Histidine pKas and subcellular pH

We have found that protein populations tend towards their most stable, on average, at the pH of the relevant subcellular environment. A couple of notes should be made about these results, which are based on predictions of pH-dependent properties from protein structures. The calculation model is simple (Debye-Hückel), based largely on geometry of the charge network. This works well for groups at the protein surface, with charge-charge interactions dominated by water, which is the vast majority of ionisable groups. In addition, the linear correlation of predicted properties with subcellular pH is good, but the fit between these properties and actual subcellular pH values falls away for the acidic vacuolar and lysosomal compartments (Figure 3(b)). We presume that in these cases the restrictions imposed by overall ionisation regions (Figure 2) prevent realisation of more acidic average values.

Taking the basic observation, of predicted maximal stability at subcellular pH, it is important to note that the bimodal distribution of individual protein pH [ΔG_FU_(min)] values means that generally each protein is not most stable at the pH of its surroundings i.e. the observed correlation relates to a sum over proteins in a particular environment. If this correlation were not observed, then in principle the folding free energy of proteins could (on average) be more stabilising at an alternate pH. Thus, with the observed correlation, the unfolded population of proteins is (on average) minimised with respect to subcellular pH. However, pH-dependent changes in ΔG_FU _for each protein, and related alteration in the F/U population, are generally small, but possibly could be significant over the subcellular population of proteins. This is a protein folding perspective on the results.

An alternative view would be to consider protonation, pH buffering and pH homeostasis, which is directly related to protein folding since ∂ΔG_FU_/∂pH αΔQ_FU _[33]. The regulation of pH is of critical importance [35] and histidine imidazoles are important components of intracellular buffering power [36]. Histidine ΔpKas underpin the relationships that we observe in the current work. In overall terms, we see that more acidic environments tend towards more acidic pH [ΔG_FU_(min)], which in turn relates to higher His pKas, more positive His ΔpKas and relatively stabilised protonated states. For example, His pKas move towards higher values, away from the subcellular pH, for acidic compartments relative to other environments. The general trend is thus to reduce the buffering power associated with His, in each location. However, this movement is small. Taken as an average value per His, the total range of pKa shift between most acidic and most basic environments is about 0.2 pH unit. Although histidine ionisation properties underlie our results, it may be that their direct contributions to proton/pH buffering are not the most important factor.

The reason that small average changes in His pKa give rise to larger changes in <pH [ΔG_FU_(min)]> (displayed schematically in Figure 2), is that the stability term includes a difference to the U state, and therefore also to the model compound pKa for His (6.3). Of key importance is ΔpKa, determined by charge interactions in the F state, so that if the model compound value changes, the overall result remains. Thus far we have discussed our results in the context of overall protein stability and pH buffering. Next we combine these aspects.

When a protein folds or unfolds it may release or take-up protons. Another way of looking at the correlations we find is that, on average and with the caveat about acidic compartments not falling directly on the line in Figure 3(b), net proton release or up-take is predicted to be close to zero upon folding or re-folding. However, this need not be the case generally, since metabolic processes leading to net changes in proton concentration are handled by the mechanisms of pH homeostasis [37].

Outside of net changes in protein folding, and without considering intrinsically unstructured proteins [38], a subset of proteins or domains will be unfolded at any given time. It is of interest to estimate the number of histidines associated with this unfolded population. Given a protein density of about 1.35 g cm^-3 ^[39], a volume fraction of around 15% for proteins in the cytoplasm [40], and an average amino acid molecular weight of 110 daltons, the cytoplasm is approximately 1.8 Molar in protein amino acids. With an estimate of histidine amino acid composition at about 2.3% [41], this gives a histidine Molarity of 0.042. If an average folded state stability is taken at around 25-30 kJ/mole [42], then about 1 in 10^5 ^domains will be unfolded, so that an approximate concentration of histidine in the unfolded state is 4 × 10^-7 ^Molar. Thus the sub-population of histidine ionisable groups that are transiently in the unfolded state could be larger than the concentration of protons. Of itself this may not be a problem, since transient changes across a compartment will average out. What could be an issue though, is whether changes in the populations of folded and unfolded histidine sites couple to local pH-dependent phenomena. This may be protein folding itself, for example with low numbers of protons at pH 7, fluctuations could impede protein refolding that is associated with proton uptake. Alternatively, the mechanisms of pH-sensing and pH homeostasis could be inappropriately activated and modulated by sufficiently large fluctuations. Whether these processes occur *in vivo *depends on the details of protein and proton diffusion properties as well as on pH-sensing mechanisms, and their response functions, all of which are unknown at the required level of detail. However, the observed tendency to average protonation changes towards zero for protein folding/unfolding, in each subcellular location, would mitigate against such processes.

## Conclusion

In this work we have asked whether the pH-dependence of organelle sub-proteomes, derived with structure-based predictions, correlates with environmental pH. We find that restrictions imposed by the composition of ionisable groups means that individual proteins have minima in pH-dependence, (the predicted pH at maximal stability), that tend to lie on either side of subcellular pH. Averages over proteins within each subcellular location though reveal a strong correlation with subcellular pH. Investigating further it is found that histidine ionisations and ΔpKas from charge interactions in the folded state underlie this correlation.

While net charge and pI also correlate with subcellular pH, and pI correlates with the pH-dependent properties reported here, the strongest relationship is found between pH-dependence (and histidine ΔpKas) and subcellular pH. Thus, while a net charge relationship with subcellular pH could be understood in terms of solubility and avoiding isoelectric aggregation, there is also the question of what lies behind the observed correlation of predicted pH-dependence and subcellular pH. At face value, it could be simply that folding stability tends towards maximal in each subcellular location. However, it is only the average that gives the correlation, rather than the stability maxima for individual proteins.

We have shown that histidine ionisation underlies the pH-dependence correlation. Further, histidine locations are key since random placement of equivalent numbers of histidines, in preserved charge backgrounds, does not reproduce the strength of correlation. This leads us to consider the proton buffering of histidine, but the differences between subcellular environments (i.e. how much histidine ΔpKas are predicted to move) are relatively small.

Finally, we address the role of histidine ionisation in protein folding/unfolding. The direct implication of our results is a prediction that proton release and proton uptake are balanced in a random subset of folding or unfolding proteins. Presumably such a balance would not be required during net protein synthesis or degradation, since the mechanisms of pH homeostasis regulate proton concentration. We speculate that a balance of proton uptake and release could play a role in guarding against activation of pH homeostatic processes during folding and unfolding fluctuations in a steady state subcellular compartment. This can be examined experimentally, with more detailed characterisation of the dynamics of pH homeostasis mechanisms, and computationally with systems level models. It will also be of interest to study the subcellular and extracellular distribution of protonation changes upon complexation. This extends to protein-protein complexation [43] and to protein-small molecule, for example the Bohr effect in hemoglobin [44].

## Methods

### Dataset

Release 55.5 of UniProtKB/Swiss-Prot [45] was searched for annotation according to the following subcellular compartments: nucleus; vacuole; cytoplasm; extracellular; lysosome; chloroplast; mitochondrion; endoplasmic reticulum; peroxisome; Golgi. Entries with uncertain keywords such as similar, potential, probable were omitted. Requiring at least one cross-reference from the Protein Data Bank (PDB) structural database [46] gave 5278 UniProt entries referencing 16311 PDB identifiers. These PDB identifiers were filtered using the PISCES server [47] for X-ray diffraction structures better than 3 Å resolution, a minimum chain length of 30 amino acids and redundancy at 90% sequence identity, yielding 3,713 protein chains. The 90% sequence filter was chosen so that identical chains would be eliminated, but allowing for amino acid variation on a common fold, since the calculated charge interactions will change with such variation. Of the 3,713 chains, we excluded those with extracellular (1,048) and chloroplast (90) annotation, as these locations present a broad pH distribution. A further 534 were annotated with more than one subcellular location and were also excluded, as well as 20 failures in the calculation scheme (for example, due to non-standard residue names). Structure-based predictions of pH-dependent properties were made for the remaining 2,021 protein chains (see Additional file 1), roughly double the number compared with previous work [20]. Figure 1 summarises this dataset.

### Calculations

Continuum models are commonly used for calculating charge interactions in biomolecules. The complexity and computational requirements of these models varies according to the accuracy with which the boundary between solute and solvent is described. In the current work, we require a relatively fast method, enabling calculations not just for many proteins, but also for a randomised dataset that is generated to evaluate the central hypothesis. A simple Debye-Hückel method is sufficient for these purposes, since most of the ionisable charge proteome is exposed to solvent, with water dominating the solvation response [29]. In earlier work in this area, it was found that the relatively simple Debye-Hückel method gave very similar results to the more computationally demanding Finite Difference Poisson-Boltzmann method [20]. A uniform relative dielectric of 78.4 and an ionic strength of 0.15 Molar were used in calculations of charge interactions. To compute ionisable group pKas [48] from these interactions, Monte Carlo sampling of protonation states was used [49]. Changes in folding energy were derived from the charge difference between folded (F) and unfolded (U) states of a protein (ΔQ_FU_) [33], with an origin set from the ionisable group contribution to the folding energy calculated at an extreme pH with the reduced sites method [48]. The following model compound pKas were used; sidechains: Asp 4.0; Glu 4.4; His 6.3; Lys 10.4; Arg 12.0; terminal groups: N-terminal 7.5; C-terminal 3.8. Cysteine and tyrosine ionisation has not been considered, since although important in certain catalytic processes, these ionisations are of less interest in a study of global charge properties around neutral pH. The unfolded state is approximated as lacking interactions between ionisable groups. While there are known to be charge interactions in the U state [50], the current work focuses on the relationship between pKas in the folded state (histidine in particular) and subcellular location. Of interest is that ionisable group interactions in the unfolded state appear to be dominated by local sequence neighbours, in part recapitulating the interactions of the folded state [29]. We trialled a simple model for pKa changes in the unfolded state [29] as a mimic for folded state pKas, examining whether the correlations observed with subcellular pH were reproduced. If this had been successful, it would have broadened the study to bypass protein structure in examining proteomes; however this trial failed, with substantially lower correlation observed between calculated properties and subcellular pH, compared with structure-based calculations.

An additional test of the Debye-Hückel-based method for calculating maximal stability was made, following a protocol established in previous work [31]. Briefly, the BRENDA enzyme database [51] was searched for text strings associated with maximal or optimal pH of stability, and these results cross-referenced with PDB entries for the same species and enzyme. The literature references retrieved from BRENDA were checked for data pertaining to a well-defined optimum, rather than a pH-range, and also for confirmation of the optimum in relation to stability as opposed to activity. Additional file 2 gives information for the 19 enzymes retrieved in this analysis. The listed criteria, and in particular the requirement for a precise species match between structure and stability data, leads to a smaller dataset than that reported previously [31]. The calculated root mean square deviation between calculated and experimental pH stability optima is 0.78 for the dataset of 19 enzymes, comparable with the value of 0.72 for the earlier work [31], and supporting the use of Debye-Hückel modelling in this study.

The quantity His/(Acid + Base) was calculated as the number of histidine residues divided by the sum of Asp, Glu, Lys and Arg residues. In many cases, averages of properties were calculated over a set of protein structures annotated with a particular subcellular location, and denoted by <> symbols.

In order to test the importance of histidine sidechains, predictions of pH dependence for proteins were made with the ionisable charges of histidine sidechains removed. Additional tests made use of proteins that conserved the number of histidine sidechain charges, but not their locations. Their positions were randomly assigned to surface atoms (within amino acids of accessible surface area > 5 Å ^2^), ensuring ionisable charges were separated by at least 3.5 Å. This distance constraint applied also to the background acidic and basic groups whose locations were unchanged. One hundred datasets of proteins (each mirroring the 2,021 proteins of the wild-type dataset) were constructed in this way, and pH-dependent features calculated. The extent of these computations required us to look at whether the full Monte Carlo sampling, to obtain pKas, could be circumvented for the properties of interest. It was established that the pKa deviations of histidine sidechains (ΔpKa = pKa - pKa [model compound]) could be estimated accurately (in relation to the Monte Carlo sampling) from summation of charge interactions at each ionisable site, at neutral pH (see Results and Discussion).

### Subcellular pH

Experimentally-determined pH values for subcellular compartments were collated from various sources in previous work [20], with an update for vacuolar pH to reflect its position in the organelle acidification pathway [52,53]: nucleus 7.7; vacuole 5.3; cytoplasm 7.3; lysosome 4.8; mitochondrion 7.5; endoplasmic reticulum 7.1; peroxisome 8.2; Golgi 6.6. There will be some uncertainty in precise values for individual locations, for example due to compartmentalisation, but the overall trend of more acidic, neutral, or more basic compartments is the key factor.

## Abbreviations

3D: three-dimensional; F: folded (protein); PDB: protein databank; pI: isoelectric point; U: unfolded (protein).

## Availability and requirements

Project name: Proteomepk

Project home page: 

The software used in this study is also available for download from: 

Operating system(s): Linux

Programming language: Fortran

License: GNU GPL

No additional restrictions for non-academic users.

## Authors' contributions

JW and PC together designed the study and wrote code for the calculations. The dataset was assembled by PC, and PC performed the calculations and most of the data analysis. JW and PC together wrote the paper.

## Supplementary Material

Additional file 1 **Dataset**. An Excel file containing the PDB and chain identifiers, and subcellular annotations, of proteins used in this work is provided. The data follow the description given in Figure 1. Click here for file

Additional file 2 **pH of max stability**. Information for the proteins used in a test of pH stability predictions. Click here for file
